# What are the differences between on-ice and off-ice side-cutting maneuver? A kinematic and electromyographic comparative analysis of ice hockey players

**DOI:** 10.3389/fbioe.2025.1692676

**Published:** 2025-11-07

**Authors:** Zijun Yu, Gengchao Bi, Weikai Wang, Ying Qin, Zihan Song, Fengyu Wu

**Affiliations:** 1 Graduate School, Harbin Sport University, Harbin, China; 2 Research Institute of Sport Science, Harbin Sport University, Harbin, China; 3 College of Sport Science and Health, Harbin Sport University, Harbin, China

**Keywords:** ice hockey, biomechanics, side-cutting maneuver, training specificity, OpenSim

## Abstract

**Introduction:**

Off-ice training is foundational for developing key physical qualities such as strength and power in ice hockey, but its biomechanical transference to on-ice performance is not well understood. This is critical, as maneuvers like side-cutting carry a high injury risk, potentially linked to environmental differences. This study aimed to compare the hip and knee kinematics and neuromuscular control strategies of elite ice hockey players during side-cutting maneuvers in on-ice versus off-ice environments, and to explore the potential injury implications associated with these biomechanical differences.

**Methods:**

Twenty elite male ice hockey players performed standardized 45° side-cutting maneuvers on and off the ice. A 12-camera motion capture system and surface electromyography (sEMG) were used to collect kinematic and muscle activation data. Biomechanical analysis was conducted using OpenSim for modeling, with one-dimensional Statistical Parametric Mapping (SPM1D) for continuous curve analysis and SPSS for discrete data points.

**Results:**

The on-ice maneuver demonstrated fundamentally different biomechanical patterns. Kinematically, athletes exhibited significantly greater hip flexion, hip abduction, and knee flexion on-ice. Most notably, a complete reversal in frontal plane knee motion was observed, shifting from a varus posture off-ice to a valgus posture on-ice. Neuromuscularly, a paradoxical strategy was revealed: while individual muscle activation (IEMG, RMS) was significantly lower on-ice, the muscle co-activation index (CI) of the knee and ankle joints was significantly higher.

**Discussion:**

The findings reveal a key adaptive trade-off: the on-ice maneuver is kinematically riskier (knee valgus) but biomechanically more efficient (lower muscle work). The increased co-activation appears to be a protective neural strategy to enhance joint stability on the low-friction surface, compensating for the vulnerable posture. This underscores a critical gap in training specificity, as off-ice patterns do not replicate on-ice stability demands. Therefore, optimal training programs must integrate exercises that simulate on-ice loading characteristics to better prepare athletes and mitigate injury risk.

## 1 Introduction

Ice hockey is a dynamic, physically demanding sport characterized by high-speed skating, abrupt stops, and frequent directional changes on a low-friction surface (Bieniec and Grabara, 2025; Buckeridge et al., 2015). The side-cutting maneuver is a crucial technique for both offense and defense, enabling players to penetrate defensive formations and create scoring opportunities. However, this maneuver also carries a significant risk of injury (Whyte et al., 2018; Aziminia et al., 2025). Executing a side-cut involves rapid deceleration and changes of direction, which place substantial biomechanical loads on the lower limbs, particularly the knee joint (Ma et al., 2024; Nedergaard et al., 2020). Consequently, epidemiological data identify the knee as one of the most commonly injured areas during on-ice hockey play, with ligamentous injuries being especially frequent (Dos'Santos et al., 2018; Havens and Sigward, 2015; Tuominen et al., 2015; Mosenthal et al., 2017).

To enhance performance and prevent injuries, ice hockey training integrates both off-ice and on-ice components (Montgomery, 1988). Off-ice training is foundational, targeting the development of key athletic qualities such as an athlete’s strength, power, and neuromuscular control. This model operates on the premise that skills and movement patterns from off-ice exercises will effectively transfer to sport-specific performance on the ice (Gerg et al., 2025; Baron et al., 2024; Evans and Gleadhill, 2024; Rice et al., 2024).

However, the efficacy of this transfer hinges on the consistency of an athlete’s movement patterns between off-ice and on-ice environments when performing actions with the same tactical goal. Significant biomechanical discrepancies between these settings could diminish the benefits of off-ice training. Worse, they might reinforce faulty movements, potentially increasing on-ice injury risk (Brown et al., 2014; Bieniec and Grabara, 2025).

On land, athletes must generate significant explosive force to overcome friction and air resistance. In contrast, on a low-friction ice surface, air resistance is the primary opposing force during gliding (Van Ingen Schenau, 1982). Fundamental differences in the support interface (shoes vs. skates) and the coefficient of friction also distinguish these environments (Rago et al., 2023). These discrepancies may compel athletes to adopt different lower limb strategies to perform the same side-cutting maneuver (Khuyagbaatar et al., 2017; Houdijk et al., 2000).

Consequently, treating off-ice training as a simple substitute for on-ice performance can overlook critical biomechanical variables and compromise the accurate assessment of athletic loads. To date, there is a lack of direct, quantitative research comparing the hip and knee kinematics of on-ice and off-ice side-cutting maneuvers among ice hockey players.

Therefore, this study aims to compare the hip and knee kinematics and muscle activation patterns of ice hockey players during on-ice and off-ice side-cutting maneuvers, and to evaluate how the observed biomechanical differences may relate to on-ice injury risk. This analysis will integrate data from high-precision motion capture, surface electromyography (sEMG), and the OpenSim 4.3 simulation platform.

Based on the fundamental biomechanical differences between high-friction land environments and the low-friction ice surface discussed previously, we formulated the following hypotheses: 1) The on-ice side-cutting maneuver will exhibit significantly different hip and knee kinematic patterns compared to the off-ice version. 2) Neuromuscular Control Hypothesis: Compared to the off-ice side-cutting maneuver, athletes performing the on-ice version will exhibit a muscle activation strategy more focused on maintaining dynamic stability.

## 2 Methods

### 2.1 Sample size calculation

The required sample size was determined *a priori* using G.Power software (Version 3.1). For a paired samples t-test, a calculation based on a large effect size (d = 0.8), an alpha of 0.05, and a power of 0.80 indicated that a minimum of 15 participants per group was required.

### 2.2 Participants

Twenty elite male ice hockey players from Harbin Sport University (age: 18.0 ± 0.7 years; height: 182.0 ± 3.8 cm; body mass: 80.8 ± 12.7 kg; training experience: 6.4 ± 1.5 years) participated in this study. The “elite” classification was defined by the formal athletic rank of National Master Sportsman, a title awarded by the General Administration of Sport of China, which represents a high level of national competitive achievement. All participants received a detailed explanation of the experimental procedures and provided written informed consent. The study was conducted in accordance with the ethical standards of Harbin Sport University and received institutional ethical approval (No. 2025042).

To be included, participants were required to be right-leg dominant, have no lower limb injuries in the 6 months preceding the study, and demonstrate proficiency in the specified side-cutting maneuver, Leg dominance was confirmed by asking each participant which leg they would preferentially use to kick a ball for maximum distance (Promsri et al., 2018).

### 2.3 Experimental equipment

This study utilized 12 high-speed infrared cameras from the Qualisys 600 series V5 (Sweden), featuring a 5-megapixel resolution and a sampling frequency of 200 Hz. Additionally, a 16-channel wireless surface electromyography system, Trigona by DELSYS (USA), was used, with a sampling frequency of 2000 Hz. Kinematic and surface electromyographic data during the side-cutting maneuver were synchronously collected using Qualisys Tracker Manager (QTM) software.

### 2.4 Data acquisition

For data collection, participants were equipped with wireless sEMG sensors and reflective markers. A 12-camera Qualisys high-speed infrared motion capture system recorded kinematic data, while a wireless sEMG system concurrently captured muscle activation. The subsequent biomechanical analysis utilized the OpenSim “gait2392” model (Xu et al., 2015), which features 12 rigid bodies, 31 degrees of freedom (DOF), and 92 muscle actuators.

Prior to testing, the skin over the target muscles was cleansed with medical alcohol to reduce impedance. Following the sEMG for the Non-Invasive Assessment of Muscles (SENIAM) project recommendations (Hermens et al., 2000), sEMG sensors were then placed on the muscle bellies, parallel to the muscle fibers. The seven muscles monitored on the right leg were the rectus femoris, vastus lateralis, vastus medialis, biceps femoris, tibialis anterior, and the medial and lateral gastrocnemius. These muscles were selected to focus on the primary movers and stabilizers of the knee and ankle joints. After sensor placement, maximal voluntary contraction (MVC) signals were recorded, Specifically, MVC for the quadriceps femoris (RF, VM, VL) was elicited via a maximal voluntary isometric contraction (MVIC) during a seated knee extension against manual resistance with the knee flexed at 90°. MVC for the biceps femoris was obtained during a maximal isometric knee flexion under similar conditions. For the tibialis anterior, participants performed a maximal isometric dorsiflexion, while the gastrocnemius (MG, LG) MVC was obtained via a maximal isometric plantar flexion. Participants held each MVIC for 5 s, and the highest value from two trials, separated by a 60-second rest, was used for normalization (Soderberg and Knutson, 2000).

The on-ice trials were conducted on a standard indoor ice hockey rink with professionally maintained ice conditions to ensure a high degree of sport-specific representativeness. The off-ice trials were performed on a high-friction sport court surface within a laboratory setting.

Participants then wore standardized form-fitting athletic apparel and their own personally fitted ice hockey skates to ensure the sport-specific representativeness of their performance. Skate sharpening profiles were not standardized and reflected each participant’s personal preference, consistent with their typical training and competition conditions. Reflective markers were affixed according to the OpenSim gait2392 marker set (Table 1). To ensure consistency, the same researcher placed all markers for all participants.

**TABLE 1:** Specific locations of the markers placed on the body.

Specific locations of the markers placed on the body		
Top of the head	Left/right anterior superior iliac spine	Left/right lateral ankle
Left/right temporal region	Left/right upper thigh	Left/right medial ankle
Sternum	Left/Right Front of Thigh	Left/right heel
Left/right acromion	Left/Right Rear of Thigh	Left/right midfoot-sup
Left/right biceps brachii	Left/right lateral knee	Left/right midfoot-lat
Left/right elbow	Left/right medial knee	Left/right toe lateral
Left/right wrist medial	Left/Right Shank of Upper	Left/right toe medial
Left/right wrist lateral	Left/Right Shank of Front	Left/right toe-tip
Sacrum	Left/Right Shank of Rear	

**FIGURE 1:**
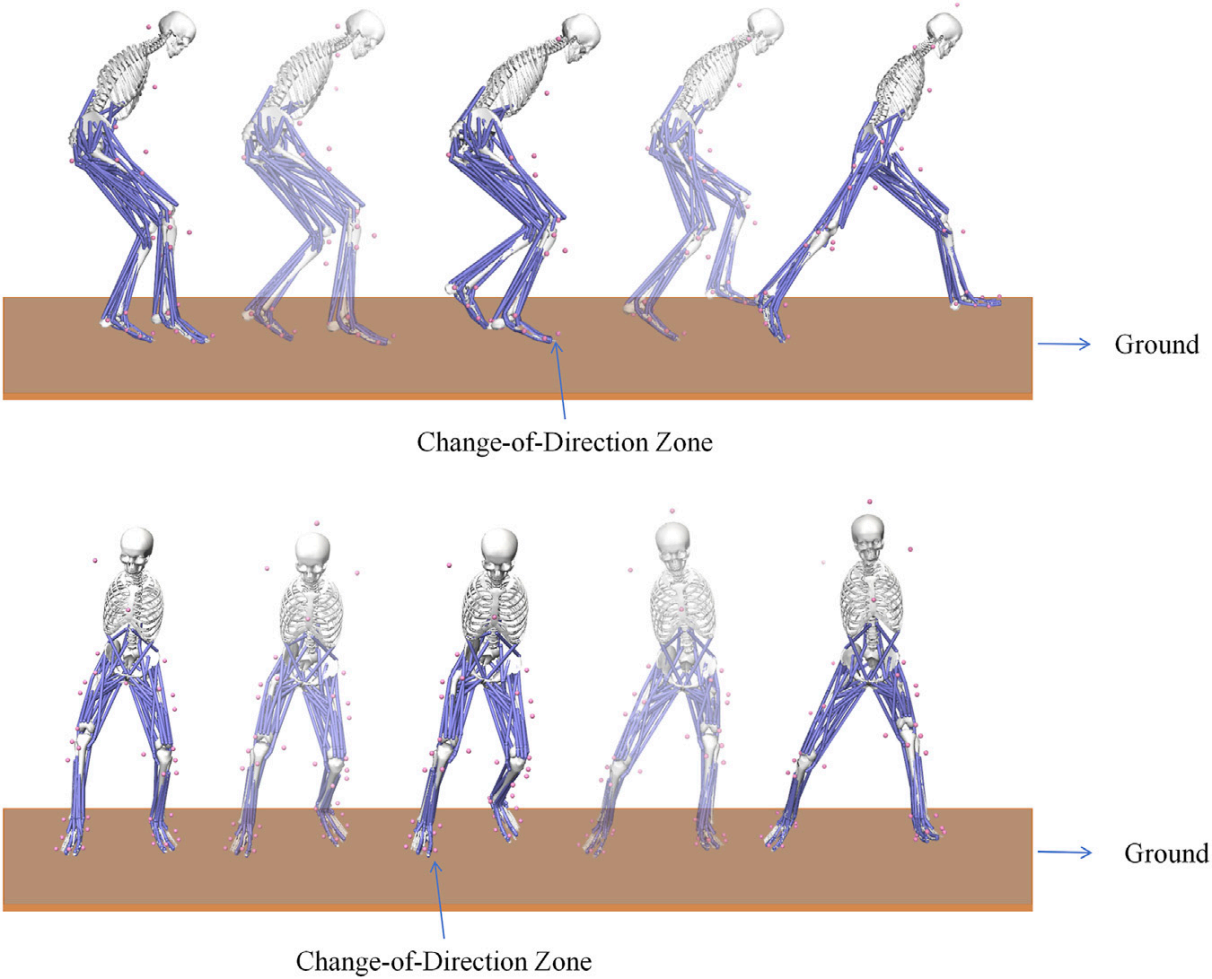
Illustration of the Off-Ice Side-Cutting Maneuver. The “Change-of-Direction Zone” indicates the marked area where participants were instructed to initiate the cut.

**FIGURE 2:**
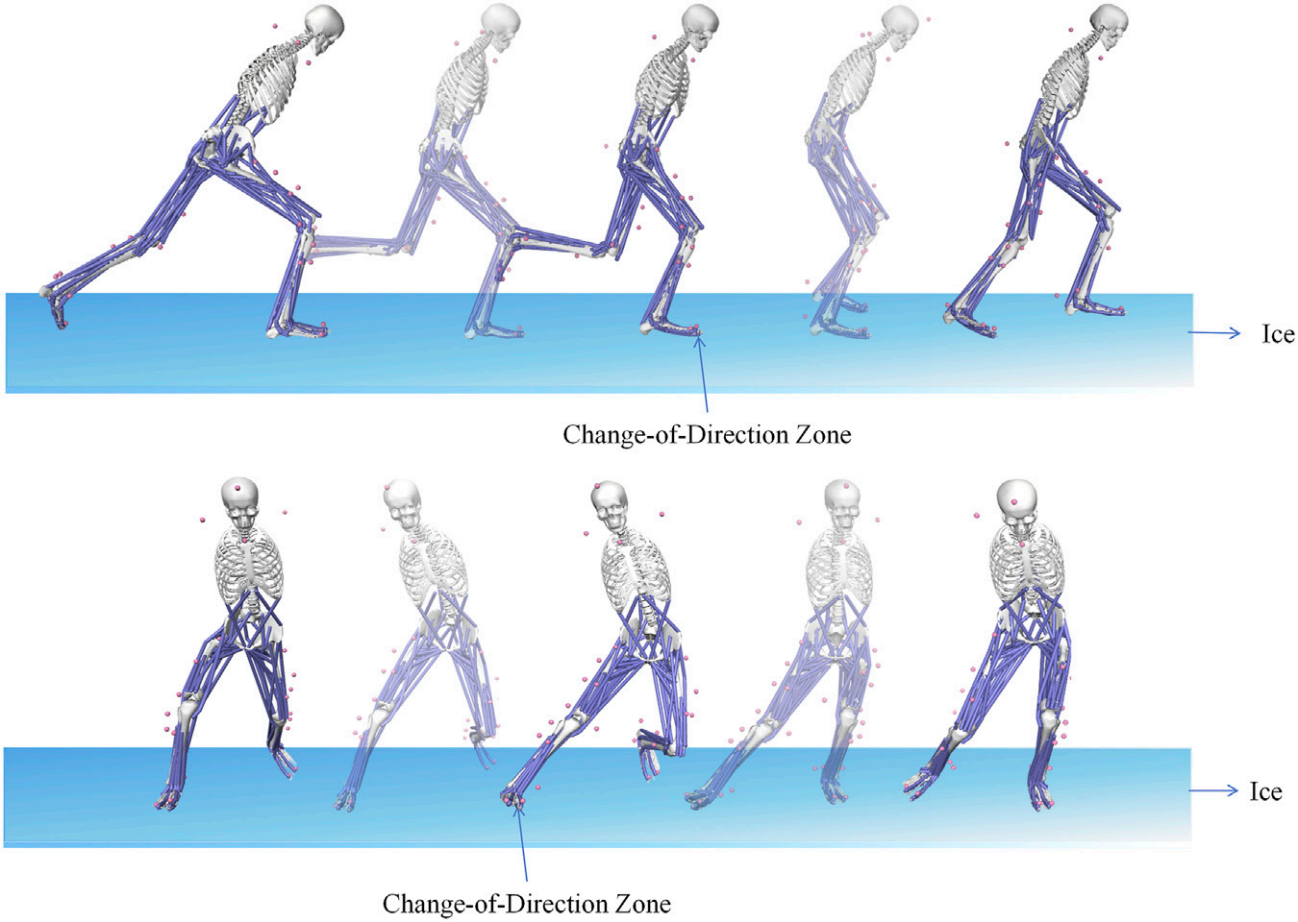
Illustration of the On-Ice Side-Cutting Maneuver. The “Change-of-Direction Zone” indicates the marked area where participants were instructed to initiate the cut.

Prior to the task, all participants completed a standardized warm-up protocol consisting of dynamic stretching and task-specific submaximal movements. After preparation, participants performed warm-up trials in both off-ice and on-ice settings to familiarize themselves with the standardized side-cutting maneuver (see Figures 1, 2). A standardized pathway was used to control the change-of-direction angle at 45°. Participants began at a starting line and maximally accelerated for 5 m. At a marked zone, they pushed off with their dominant (right) leg to cut left. An exit path, angled at 45° to the initial approach, guided their movement for an additional 2 m. The course was clearly marked to ensure angular consistency. Each participant completed five successful trials, with a 60-second rest period between each trial to minimize the effects of fatigue. The trials were then averaged for analysis to minimize error.

### 2.5 Simulation process and data processing

Raw kinematic data were exported from QTM in.c3d format and imported into Matlab R2021a. The data were then converted to a.trc file format using the conversion module in OpenSim 4.3.

The biomechanical simulation followed a standard workflow. First, the “gait2392” model was scaled to match each participant’s anthropometry. Next, an Inverse Kinematics (IK) analysis was performed on the.trc files to compute joint angles. Finally, the Static Optimization (SO) algorithm estimated muscle activations. These estimated activations were then compared against the recorded sEMG data for model validation.

Raw sEMG data were processed in Matlab R2021a. The processing pipeline included demeaning the signal, applying a 20–480 Hz Butterworth band-pass filter, and then using a 20 Hz low-pass filter to create the signal envelope. In Origin2018, each movement cycle was time-normalized to 100%. The resulting envelopes from all trials were averaged to generate a representative activation pattern for each muscle, normalized to its MVC.

To facilitate a more granular analysis, the side-cutting maneuver was partitioned into distinct phases for both on-ice and off-ice conditions. For the off-ice condition, the ground contact time of the stance leg was divided into two functional phases consistent with previous cutting analysis literature: the Weight Acceptance Phase, from initial foot contact to maximal knee flexion, and the Push-off Phase, from maximal knee flexion to toe-off (Havens and Sigward, 2015). For the on-ice condition, the maneuver was partitioned into three consecutive phases based on key kinematic events: the COM Transfer Phase, from the initial significant decrease in forward center of mass (COM) velocity to the moment of minimum vertical COM height; the Push-off Phase, from minimum vertical COM height to the point at which the push-off skate blade loses contact with the ice (blade-off); and the Glide & Re-acceleration Phase, from blade-off to the initial contact of the contralateral skate.

The outcome measures for this study included:

Kinematic Variables:Kinematic analysis focused on the hip and knee joints. Variables included hip flexion/extension, abduction/adduction, and internal/external rotation, as well as knee flexion/extension and varus/valgus angles. The ankle joint was excluded from the kinematic analysis because the rigid construction of modern ice hockey skates significantly restricts its natural range of motion, providing data with limited interpretive value (Campanelli et al., 2015).

sEMG variables: sEMG variables were calculated for seven muscles: rectus femoris, vastus lateralis, vastus medialis, biceps femoris, tibialis anterior, medial gastrocnemius, and lateral gastrocnemius. The primary measures were the MVC-normalized root mean square (RMS) and integrated EMG (IEMG). Additionally, a muscle co-activation index (CI) was calculated using the formula (Bi et al., 2024): $\text{CI}=\frac{\text{EMGantagonist}}{\text{EMGagonist}+\text{IEMGantagonist}}*100\%$ . For the knee joint, the quadriceps (rectus femoris, vastus lateralis, vastus medialis) were defined as the agonist and the biceps femoris as the antagonist. For the ankle joint, the tibialis anterior was the agonist, and the gastrocnemius (medial and lateral heads) served as the antagonist.

### 2.6 Model validation

**FIGURE 3:**
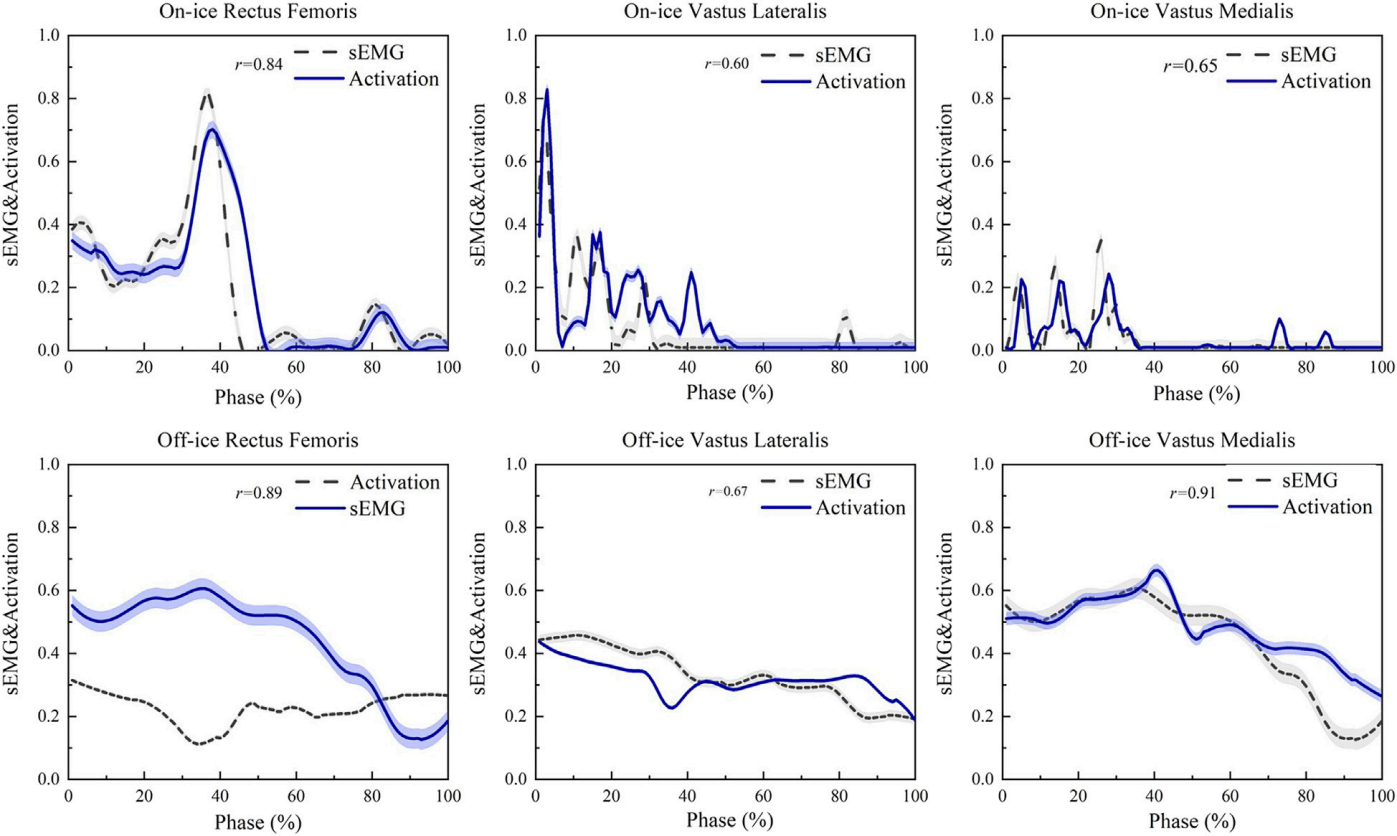
Validation of the OpenSim Model: A Comparison Between Experimental sEMG Envelopes and Model-Computed Muscle Activations. This figure shows a representative comparison between model-computed muscle activations (dashed lines) and the corresponding experimental sEMG envelopes (solid lines). The rectus femoris, vastus medialis, and biceps femoris are shown as representative examples of the primary knee extensors and flexors. A strong correspondence in activation patterns was observed across all seven measured muscles. Data are time-normalized to the stance phase (100%) and amplitude-normalized.

The OpenSim model was validated by comparing the model-computed muscle activations against the experimental sEMG envelopes. In addition to a qualitative comparison of the curve patterns Figure 3. A quantitative analysis was performed by calculating the Pearson correlation coefficient (r) between the two sets of curves for each muscle and each participant. The quantitative analysis revealed good correspondence, with the average correlation coefficients for all seven measured muscles consistently exceeding 0.60. This strong correspondence provides confidence in the reliability of the OpenSim simulation results.

### 2.7 Statistical analysis

Statistical analyses were performed using SPSS 27.0 (IBM Corp., Armonk, NY, USA). Before analysis, all discrete data (peak joint angles and sEMG variables) were assessed for normality using the Shapiro-Wilk test, and results are presented as mean ± standard deviation (Mean ± SD). Paired samples t-tests (two-tailed) were used to compare outcomes between conditions, with the LSD method applied for post-hoc tests. For continuous data, one-dimensional Statistical Parametric Mapping (co-1D) was conducted in Matlab R2021a to analyze the joint angle curves across the entire movement cycle. For all statistical tests, the significance level was set at *P* < 0.05.

## 3 Results

### 3.1 Kinematic results

#### 3.1.1 Peak joint angles

As shown in Table 2, the on-ice side-cutting maneuver produced significantly different hip and knee kinematics in the sagittal and frontal planes compared to the off-ice condition. Specifically, on-ice performance resulted in greater hip flexion (*P* < 0.01), knee flexion *(P* < 0.01), and hip abduction (*P* < 0.01). The most pronounced difference occurred at the knee, which shifted from a varus position off-ice to a valgus position on-ice (*P* < 0.01). In contrast, no significant difference was observed in hip internal rotation between the two environments (*P* = 0.92).

**TABLE 2:** Comparative analysis of peak hip and knee joint angles between on-ice and off-ice side-cutting maneuvers (mean ± SD, °).

Joint	Movement	Off-ice	On-ice	t-value	*P*
Hip	Flexion (+)/extension (−)	43.39 ± 3.51	65.83 ± 11.48	5.91	**<0.01**
	Adduction (+)/abduction (−)	−27.86 ± 4.28	−39.28 ± 3.90	−6.23	**<0.01**
	External (+)/internal (−)rotation	−19.54 ± 2.61	−19.35 ± 5.30	0.10	0.92
Knee	Extension (+)/flexion (−)	−63.22 ± 4.81	−75.18 ± 6.21	−4.82	**<0.01**
	Varus (+)/valgus (−)	15.88 ± 1.53	−14.10 ± 0.96	−52.66	**<0.01**

Bold values indicate statistically significant differences (P < 0.05) between on-ice and off-ice conditions.

#### 3.1.2 Full-cycle joint kinematic curves

**FIGURE 4:**
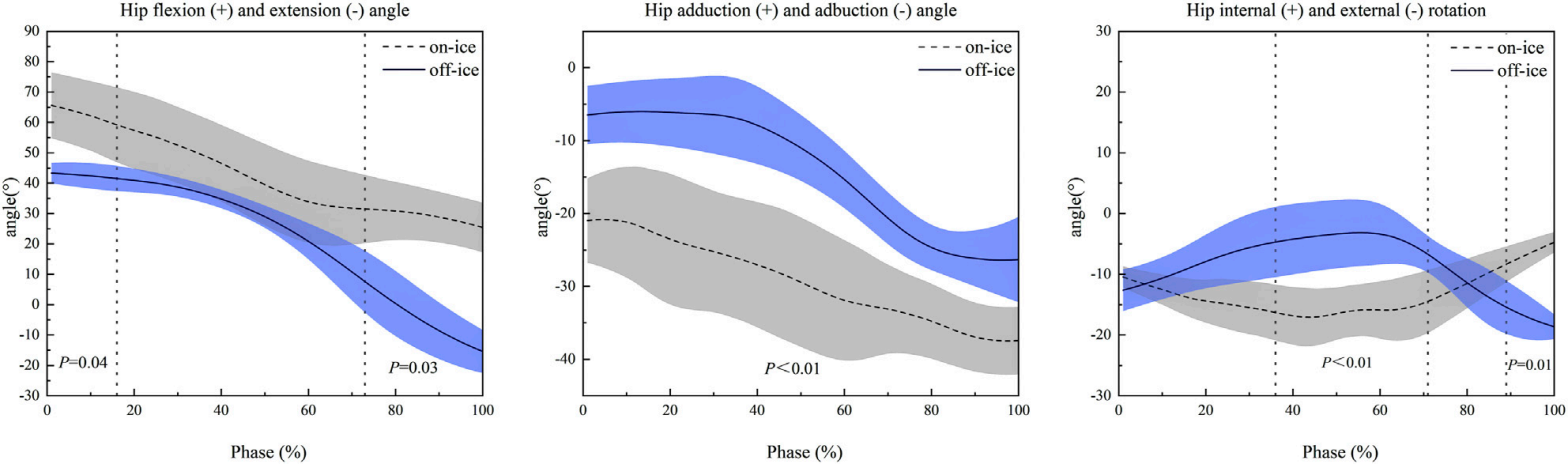
Comparison of Hip Joint Kinematics in Three Planes of Motion During On-Ice vs. Off-Ice Side-Cutting Maneuvers. Shaded areas represent one standard deviation. The x-axis is the time-normalized stance phase (100%). Positive values on the y-axis represent flexion, adduction, and external rotation, respectively. Dotted vertical lines indicate the time points or intervals where significant differences (as indicated by the p-value) were observed between the two conditions.

Figure 4 displays the continuous hip joint angle curves during the side-cutting support phase for both conditions. The SPM1D analysis revealed significant differences between on-ice and off-ice maneuvers across all three planes of motion. In the sagittal plane, hip flexion was significantly greater on-ice during the initial part of the stance phase (1%–16%, *P* = 0.04), occurring within the on-ice COM Transfer Phase and the off-ice Weight Acceptance Phase. This difference re-emerged during the final portion of the movement (73%–100%, *P* = 0.03), occurring within the on-ice Glide & Re-acceleration Phase and the off-ice Push-off Phase. In the frontal plane, hip abduction was significantly greater on-ice throughout the entire support phase (0%–100%, *P* < 0.01). Finally, in the transverse plane, significant differences in hip internal/external rotation emerged primarily during the mid-stance period (36%–71%, *P* = 0.001), falling within the on-ice Push-off Phase and the late Weight Acceptance/early Push-off phases off-ice, and again during the terminal portion of the on-ice Glide & Re-acceleration Phase (89%–100%, *P* = 0.01).

**FIGURE 5:**
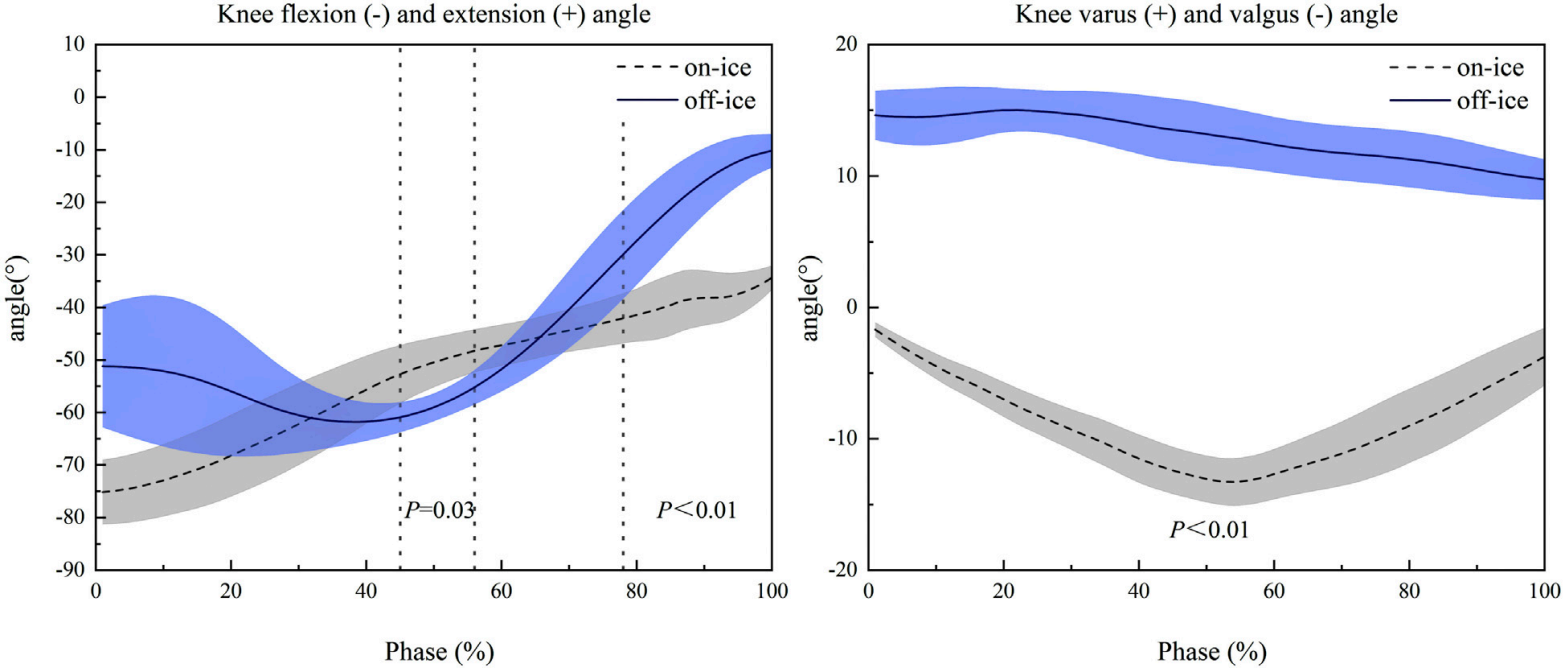
Comparison of Knee Joint Kinematics in the Frontal and Sagittal Planes During On-Ice vs. Off-Ice Side-Cutting Maneuvers. Shaded areas represent one standard deviation. The x-axis is the time-normalized stance phase (100%). Positive values on the y-axis represent extension and varus, respectively. Dotted vertical lines indicate the time points or intervals where significant differences (as indicated by the p-value) were observed between the two conditions.

As shown in Figure 5, kinematic analysis of the knee joint revealed significant pattern differences between the on-ice and off-ice conditions. In the frontal plane, the movement patterns were diametrically opposed. The knee maintained a varus position throughout the off-ice maneuver, whereas it remained in a valgus position during the on-ice maneuver. This difference was statistically significant across the entire movement cycle (1%–100%, *P* < 0.01). In the sagittal plane, significant differences in knee flexion occurred in two distinct phases. During mid-stance (45%–56%, *P* = 0.03), falling within the on-ice Push-off phase and the off-ice transition between Weight Acceptance and Push-off, on-ice knee flexion was significantly less than off-ice. Conversely, during the final portion of the on-ice Glide & Re-acceleration Phase (78%–100%, *P* < 0.01), on-ice knee flexion was significantly greater compared to the end of the off-ice Push-off Phase.

### 3.2 sEMG results

Table 3 shows that neuromuscular control strategies differed significantly between the two environments. Generally, muscle activation was lower on-ice. The IEMG and RMS values for the rectus femoris, vastus medialis, medial and lateral gastrocnemius, and tibialis anterior were all significantly reduced compared to the off-ice condition (*P* < 0.05). In contrast, activation of the vastus lateralis and biceps femoris did not differ significantly between environments (*P* > 0.05). Despite this overall decrease in individual muscle activity, a key opposing trend emerged. The CI for both the knee (*P* < 0.01) and ankle (*P* = 0.03) joints was significantly higher during on-ice side-cutting.

**TABLE 3:** Comparison of selected lower limb muscle activation and joint co-activation indices (CI) between on-ice and off-ice side-cutting maneuvers (mean ± SD).

Location	Parameter	Off-ice	On-ice	t-value	*P*
Rectus femoris	IEMG	0.25 ± 0.01	0.13 ± 0.01	−18.45	**<0.01**
	RMS	0.33 ± 0.01	0.21 ± 0.01	−14.56	**<0.01**
Vastus medialis	IEMG	0.18 ± 0.02	0.15 ± 0.01	−2.70	**0.04**
	RMS	0.25 ± 0.01	0.22 ± 0.01	−3.61	**0.01**
Vastus lateralis	IEMG	0.15 ± 0.02	0.12 ± 0.01	−2.25	0.07
	RMS	0.19 ± 0.02	0.19 ± 0.02	−0.32	0.75
Biceps femoris	IEMG	0.191 ± 0.01	0.19 ± 0.02	−0.10	0.92
	RMS	0.26 ± 0.02	0.25 ± 0.02	−0.33	0.75
Lateral gastrocnemius	IEMG	0.24 ± 0.03	0.13 ± 0.01	−7.70	**<0.01**
	RMS	0.31 ± 0.02	0.20 ± 0.02	−9.55	**<0.01**
Medial gastrocnemius	IEMG	0.16 ± 0.02	0.01 ± 0.02	−5.83	**<0.01**
	RMS	0.25 ± 0.02	0.16 ± 0.02	−7.48	**<0.01**
Tibialis anterior	IEMG	0.15 ± 0.03	0.12 ± 0.01	−2.43	**0.04**
	RMS	0.26 ± 0.02	0.18 ± 0.01	−8.72	**<0.01**
Knee joint	CI	0.25 ± 0.01	0.29 ± 0.02	3.79	**<0.01**
Ankle joint	CI	0.32 ± 0.02	0.34 ± 0.01	2.57	**0.03**

Bold values indicate statistically significant differences (P < 0.05) between on-ice and off-ice conditions.

## 4 Discussion

### 4.1 Kinematic adaptations in elite athletes

#### 4.1.1 Adaptive strategies of the hip joint

A key finding of this study is that on-ice side-cutting requires significantly greater hip flexion and greater hip abduction compared to the off-ice maneuver. This postural adjustment, which supports our initial hypothesis, appears to be a functional adaptation to the low-friction environment. Lowering the body’s center of gravity via greater hip flexion is a crucial strategy for enhancing stability on a thin skate blade (Liu et al., 2022; Alentorn-Geli et al., 2009). Simultaneously, greater hip abduction is necessary to achieve the body lean required to engage the skate’s inside edge, which generates the centripetal force for the turn. This contrasts sharply with off-ice cutting, where athletes can rely on ground friction and do not need such an exaggerated lean or lowered center of gravity (Purevsuren et al., 2018; Van Ingen Schenau, 1982).

The SPM1D analysis revealed that the difference in hip abduction persisted throughout the entire support phase (0%–100%). This finding suggests a fundamental divergence in force production strategies. On-ice, athletes must maintain a large abduction angle to continuously “grip” the ice with the skate’s edge. In contrast, the off-ice maneuver relies on a smaller range of abduction, leveraging ground friction instead. More interestingly, significant differences in hip internal/external rotation emerged primarily during the critical period for generating change-of-direction force, falling within the on-ice Push-off Phase and the late Weight Acceptance/early Push-off phases off-ice (36%–71%), which is the critical period for generating change-of-direction force. This timing suggests that, on ice, athletes may use more active hip rotation to drive the turn, potentially as a compensatory strategy for the restricted ankle mobility imposed by the skate.

#### 4.1.2 Kinematic reversal of the knee joint and its injury implications

The most significant finding of this study is the diametrically opposed knee kinematics in the frontal plane: a knee varus posture during off-ice cutting versus a knee valgus posture on-ice. This reversal is not just a key difference in movement strategy but also carries critical implications for understanding injury mechanisms.

This dramatic shift from a knee varus off-ice to a knee valgus on-ice highlights the fundamental biomechanical differences between the maneuvers. The divergence is primarily driven by the unique demands of the on-ice environment. To generate centripetal force on a low-friction surface, an athlete must lean inward and push off with the skate’s inside edge (Rago et al., 2023). This action inherently forces the support knee into a valgus alignment to execute the turn. In contrast, land-based cutting relies on friction between the shoe and the ground, which allows the athlete to remain more upright. This keeps the knee in a more stable varus or neutral position.

The knee valgus pattern observed during on-ice side-cutting provides a direct kinematic explanation for the high incidence of medial collateral ligament (MCL) injuries in ice hockey (Naqvi and Sherman, 2025). Abnormal joint motions, such as knee valgus, are not only linked to acute injuries but may also contribute to long-term degenerative joint disease (Hawker, 2023). Knee valgus is a primary mechanism for MCL injury because it places excessive tension on the ligament (Lucidi et al., 2024; Braaten et al., 2022; Miyaji et al., 2022; Xu et al., 2025). The on-ice side-cutting maneuver, by continuously generating valgus loads, repeatedly stresses the medial structures of the knee. This repetitive loading inherently increases the cumulative risk of MCL injury.

The opposing knee kinematics—varus off-ice versus valgus on-ice—highlight a critical disconnect between off-ice training and on-ice performance. While traditional off-ice training builds a vital foundation of strength and fitness, its biomechanical patterns do not fully replicate the specific demands of skating. Addressing this specificity gap is crucial for injury prevention. Future off-ice training should therefore evolve beyond foundational conditioning to include exercises that better simulate the unique valgus loading patterns of on-ice movements. For instance, exercises such as slideboard lunges, banded lateral walks, and single-leg rotational jumps could help athletes develop the specific strength and neuromuscular control required to manage these on-ice demands. This approach could enhance training transfer and more effectively mitigate the risk of sport-specific injuries.

### 4.2 Neuromuscular control characteristics

At the neuromuscular level, this study revealed a seemingly paradoxical finding. While the activation of most individual lower limb muscles (IEMG and RMS) was significantly lower on-ice, the CI of the knee and ankle joints was significantly higher. This suggests a strategic shift in neuromuscular control between environments. The on-ice condition appears to favor a strategy that prioritizes joint stability (via higher co-activation) over maximizing force production from individual muscles. This finding strongly supports our neuromuscular control hypothesis.

#### 4.2.1 Decreased muscle activation: a reflection of efficiency in gliding kinematics

The reduced muscle activation on ice is a direct consequence of the kinematic shift toward a more biomechanically efficient gliding motion (Kaartinen et al., 2024; Felser et al., 2016; Higgins et al., 2008). On ice, athletes adopt a deep, flexed posture to lower their center of gravity for stability. In this position, turning relies more on efficiently redirecting momentum through the skate-ice interaction—a smooth “glide”—rather than on the explosive muscular “push-off” needed to overcome friction on land (Van Ingen Schenau, 1982). This strategic shift diminishes the need for high force output. Consequently, the lower activation of primary movers like the rectus femoris and gastrocnemius suggests that the net muscular work required for on-ice side-cutting is significantly less.

#### 4.2.2 Increased Co-activation: a necessary response to risky kinematics

In stark contrast to the lower activation of individual muscles, the co-activation index of the knee and ankle joints increased significantly on ice. Co-activation—the simultaneous contraction of agonist and antagonist muscles—is a neuromuscular strategy the body uses to increase joint stiffness and enhance stability in unpredictable environments (Gribble et al., 2003). Executing a high-speed cut on a thin skate blade presents precisely such a challenge, demanding exceptional balance and joint control. The observed increase in co-activation is therefore a logical adaptive strategy to stabilize the lower limbs on the unstable ice surface.

By integrating kinematics with electromyography, this study reveals a key adaptive trade-off in on-ice side-cutting (Kelly and Secomb, 2024; Franca et al., 2022; Ge et al., 2025). The unique constraints of skating compel athletes into a kinematically vulnerable knee valgus posture, placing medial structures like the MCL at increased injury risk. This neuromuscular adaptation increases joint stiffness, providing the dynamic stability required to shield passive structures from excessive strain and ensure effective force transmission (Myer et al., 2005; Hua et al., 2024; Granacher et al., 2006). While this co-activation is a necessary protective mechanism, its potential long-term effects, such as increased joint contact stress, warrant further investigation to fully understand its impact on athletes’ joint health.

### 4.3 Limitations

This study has several limitations that should be considered. 1) Lack of Kinetic Data: The absence of on-ice kinetic data precluded a direct analysis of the underlying forces. 2) Limited Task Representativeness: The use of a single, standardized 45° cutting task limits the findings’ representativeness of dynamic, in-game situations. This standardized maneuver does not fully capture the unpredictable nature of actual gameplay. 3) Sample Homogeneity: generalizability is limited as the sample consisted exclusively of young, elite male athletes. Our sample consisted exclusively of young, elite male athletes. Consequently, the findings may not be generalizable to female athletes or players of different ages and skill levels. 4) Ankle Kinematics: Ankle kinematics were excluded; while we initially cited the skate’s rigidity, we acknowledge our standard OpenSim model and marker set were not optimized to capture the subtle ankle movements demonstrated in previous literature (Upjohn et al., 2008; Wu et al., 2015).

### 4.4 Future directions

Future research should prioritize acquiring direct on-ice kinetic data to elucidate the underlying forces of skating maneuvers. The neuromuscular analysis, in particular, requires expansion; our analysis was limited in both scope and depth as we excluded key hip muscles (e.g., gluteals) and were confined to basic time-domain sEMG features. Future work should therefore incorporate a broader muscle selection and employ more advanced analytical techniques, such as frequency-domain or machine learning analyses, to provide a more comprehensive understanding of the neuromuscular control of this maneuver (Xu et al., 2025). Finally, to improve generalizability, studies should incorporate more diverse participant samples and a wider range of dynamic, unpredictable tasks that better reflect actual gameplay.

## 5 Conclusion

This study compared the hip and knee kinematics and neuromuscular control of ice hockey players during on-ice versus off-ice side-cutting to reveal biomechanical adaptations. The results show fundamental differences in both movement and muscle activation patterns between the two environments. A key finding of this study is the significant increase in lower limb muscle co-activation on the low-friction ice surface. This appears to be a functional neural adaptation that increases joint stiffness to ensure dynamic stability during high-speed turns. This highlights the principle of training specificity: while off-ice training builds foundational strength, it fails to replicate the unique stability demands and muscle recruitment of on-ice performance. Therefore, optimal training systems must integrate off-ice general conditioning with on-ice practice to refine sport-specific neuromuscular control.

## Data Availability

The raw data supporting the conclusions of this article will be made available by the authors, without undue reservation.
